# A Novel Swine Model for Inducing Functional Tricuspid Valve Regurgitation

**DOI:** 10.1007/s12265-024-10510-0

**Published:** 2024-05-16

**Authors:** Dawei Lin, Peng Zhang, Yongchao Zhao, Wanjiao Chen, Dandan Chen, Xiaochun Zhang, Daxin Zhou, Junbo Ge

**Affiliations:** 1 Department of Cardiology, Zhongshan Hospital, Fudan University, Shanghai, China; 2 Department of Cardiology, Shanghai Fifth People’s Hospital, Fudan University, Shanghai, China

**Keywords:** Functional tricuspid regurgitation, Catheter radiofrequency ablation, Swine model

## Abstract

Functional tricuspid regurgitation (FTR) is the most common TR, although experimental models to effectively study it are scarce; therefore, this study aimed to establish a robust experimental swine model. A swine FTR model was developed using radiofrequency ablation, atrial septostomy, and right atrial volume overload. The baseline and follow-up echocardiography was performed to evaluate the progression FTR and changes in the heart. Autopsy was employed to verify the anatomy of tricuspid valve. One-month post intervention, among the subjects, one (8.3%) exhibited severe FTR, eight (66.7%) exhibited moderate TR, and three (25%) exhibited mild FTR. Each pig developed an atrial septal defect (diameter, 1.5 ± 0.5 cm). The tricuspid annular diameter significantly increased with enlargement of right heart (*P* < 0.05). No significant difference was found on left heart size and mitral regurgitation. We successfully developed a novel swine FTR model, providing a reliable and effective platform for further research on FTR.

**Graphical Abstract:**

The procedure to induce the FTR model on swines

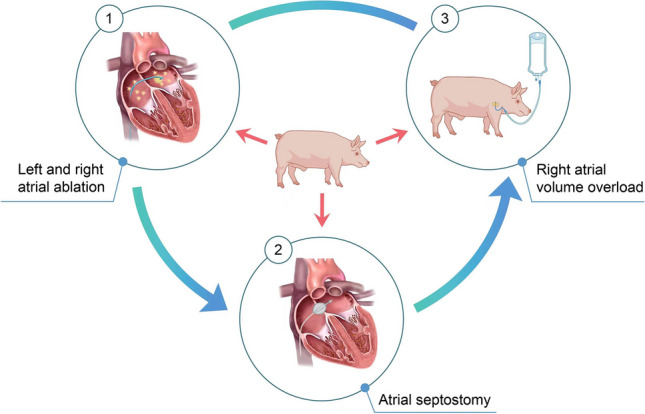

## Introduction

Tricuspid regurgitation (TR) is a prevalent cardiovascular condition affecting a substantial global population, with estimates surpassing 70 million individuals, and is associated with worse outcomes if left untreated [1]. Despite its clinical significance, TR has traditionally received less attention than other cardiac valve issues. Notably, the majority of TR cases, with its incidence ranging from 70 to 90%, are categorized as “functional” TR (FTR), resulting from pulmonary artery hypertension, left heart failure, atrial fibrillation, and so on, carrying an adverse prognosis, which is related to its severity[2, 3]. As the trend toward early intervention and technology continues, transcatheter tricuspid valve intervention has emerged as a promising therapeutic approach for FTR [4, 5]. However, to facilitate comprehensive pathophysiological investigations and preclinical studies on device development, the availability of a reliable and reproducible experimental model that effectively simulates FTR is of utmost importance.

Currently, the development of FTR animal models poses technical challenges, resulting in limited availability of methods. The tachycardia-induced cardiomyopathy model has demonstrated significant FTR. However, the biventricular model in end-stage heart failure is limited in simulating FTR in patients without left heart failure [6]. In vitro FTR models, such as the swine heart bench model, have shown limitations in replicating the complex pathophysiology of FTR [7]. Basing on experience from several investigations on how to establish an FTR swine model by us, right atrium (RA) dysfunction should be a feasible and effective way to induce FTR, while it is hard to achieve using just a single modeling method, such as tachycardia and septal fistula.

Therefore, in this study, we aimed to established a novel swine FTR model using the combination of several modeling method including RA and left atrium (LA) ablation, atrial septostomy, and right heart overload. It would offer a reliable and reproducible approach for inducing FTR, thereby addressing the limitations of existing animal models. This innovative model may contribute to an improved understanding and management of FTR.

## Methods

### Ethical Approval

All animal experiments conducted in this study were performed in strict compliance with the institutional policies and guidelines governing the ethical use of animals in research. The study protocol was approved by the Institutional Animal Care and Use Committee of Zhongshan Hospital, Fudan University.

### Preparation of the Animals

From November 2022 to January 2024, 12 healthy female adult swine (Yorkshire pigs weighing, 80.5 ± 7.5 kg) were enrolled in this study. All the subjects underwent the combination of three modeling methods: ablation, atrial septostomy, and right heart overload. After intubation, mechanical ventilation was initiated, and propofol (2–5 mg/kg IV) was administered. Anesthesia was maintained with inhaled isoflurane at concentrations of 1–2.5%, and fentanyl (5–20 mcg/kg/min) was used for sedation. Percutaneous punctures were performed in the femoral artery and veins to insert arterial and venous pressure lines for continuous monitoring. Following the induction of anesthesia, intravenous enrofloxacin (2.5 mg/kg; Bayer Health Care LLC, Shawnee Mission, KS, USA) was administered, and an additional dose was administered postoperatively to prevent infection. After completing the intervention procedures, the pigs were carefully monitored until they regained consciousness and remained in a stable hemodynamic state. The pressure lines and chest tubes were removed, and the pigs were returned to the animal house for recovery. For 3 consecutive days, each pig received oral cefuroxime (500 mg twice daily) as a prophylactic antibiotic treatment.

### Surgical Technique to Create TR

#### Ablation

**Fig. 1:**
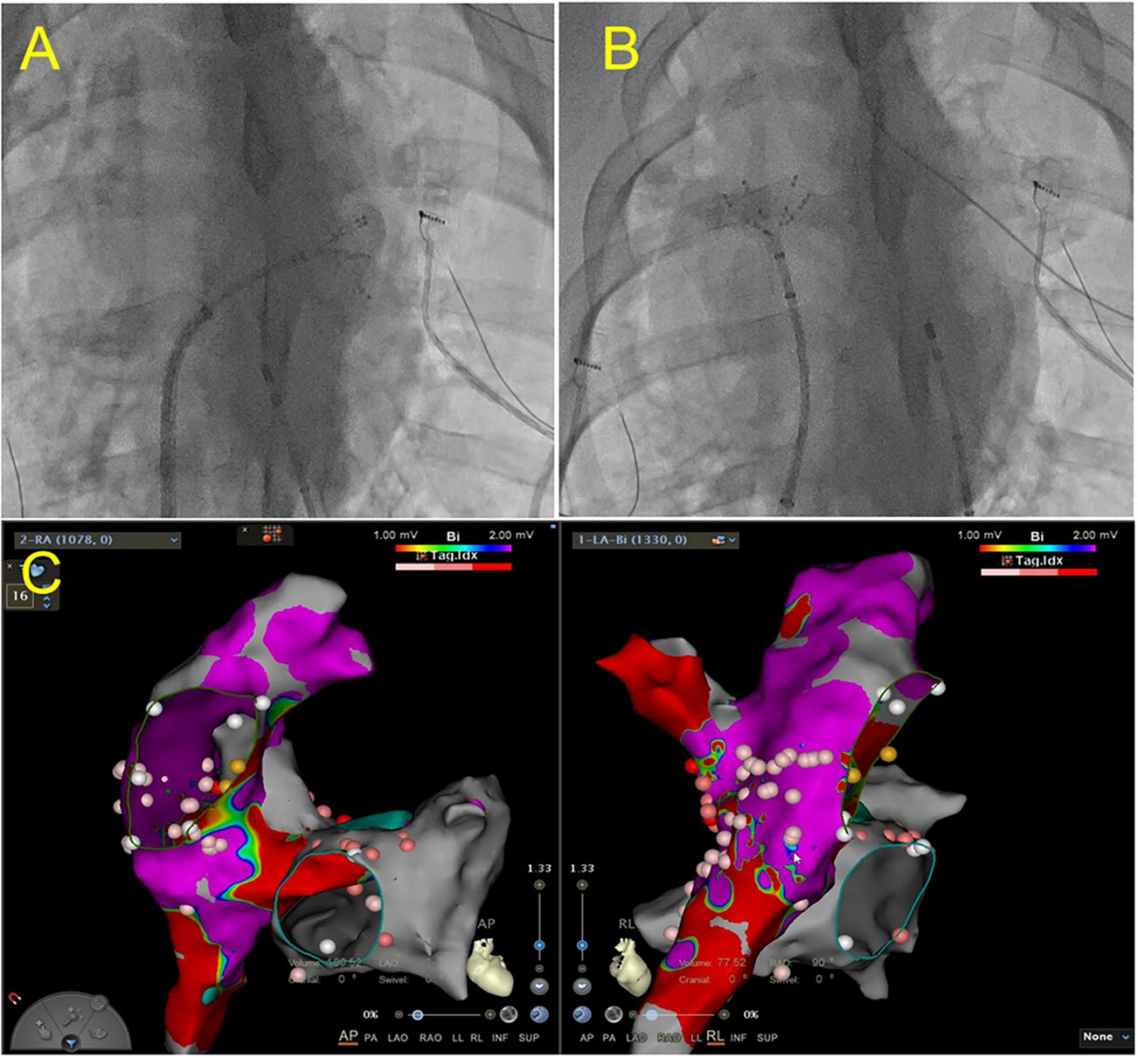
Left and right atrium ablation. **A** Ablation of left atrium. **B** Ablation of right atrium. **C** Radiofrequency ablation location in left and right atrium

We did RA and LA endocardial ablation to make a scar which would impair the atrium function and induce premature atrial beats. In order to induce RA hemodynamic disorders, endocardial ablation was employed especially on most area of RA. Together with RA ablation, LA ablation was done to help induce premature atrial beats. Transcatheter procedures were performed under general anesthesia. Ablation was performed using a commercial bipolar device (Cardioblate BP2; Medtronic Inc., Minneapolis, MN, USA) with three-dimensional electroanatomic mapping guidance. Mapping was performed with integrating atrium computed tomography images to delineate the anatomy. The atrial septal puncture was performed to access the catheters. Once the electrodes were located in the chamber and the catheters were positioned approximately at the mid-cavity, they were moved until they contacted the endocardial surface, after which ablation was performed (Fig. 1). The ablation was employed in > 80% of the right atrial endocardium, and < 30% of left atrial endocardium, resulting in premature atrial beats, which were documented using an electrocardiogram monitoring system. Data from each system were analyzed using investigational software (Conduct NT; CD Leycom, Inc.).

#### Atrial Septostomy

**Fig. 2:**
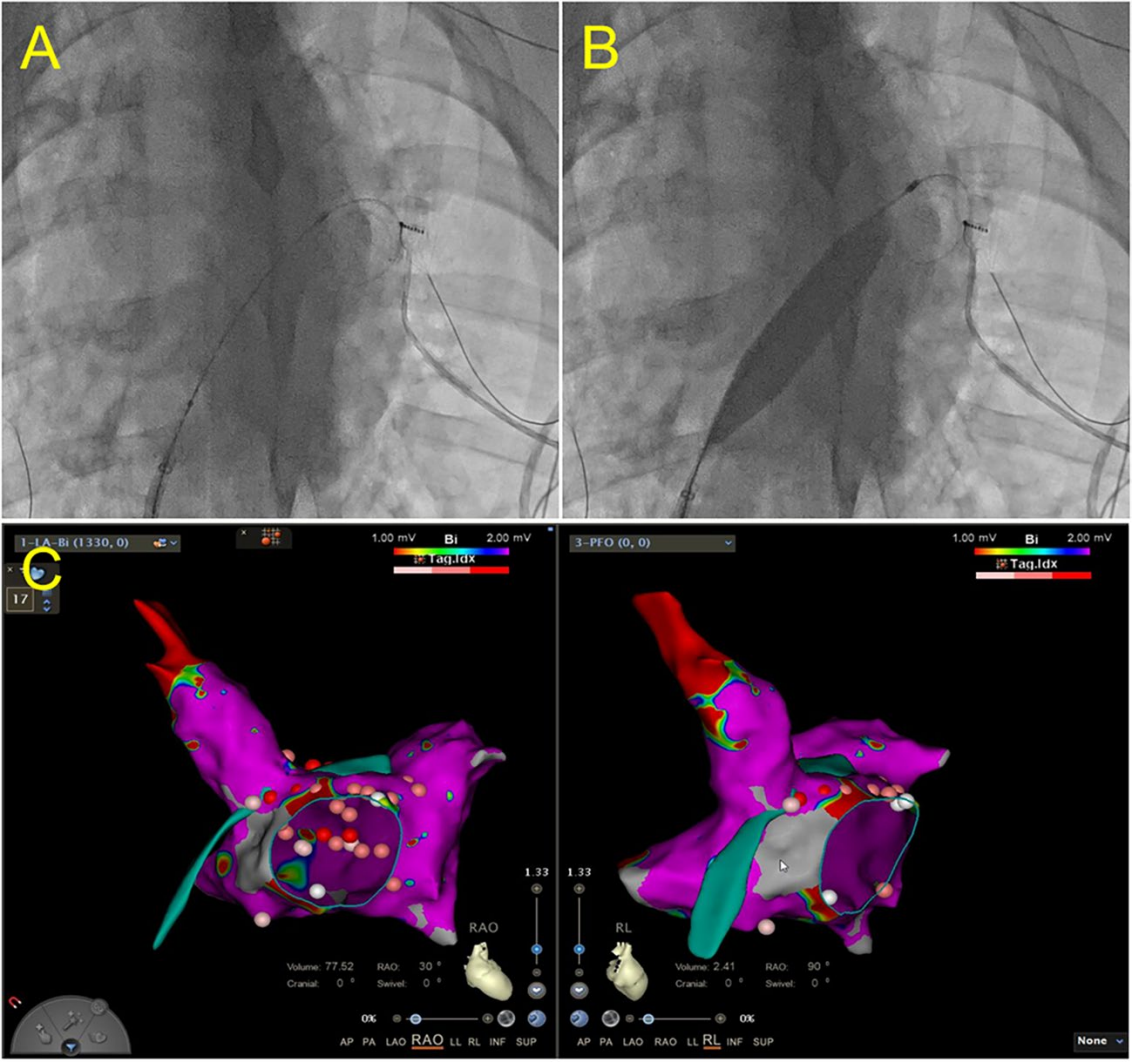
Interatrial fistulization. **A** Before the dilation of balloon. **B** Dilation of balloon. **C** Radiofrequency ablation location in patent foramen ovale

A left-to-right shunt was created through transseptal puncture, radiofrequency ablation, and balloon dilation, creating a stable interatrial communication which allowing blood flow from the LA to the RA and increase RA volume. To create a defect suitable for ablation, an atrial septal puncture was performed. To enlarge the defect further, a transcatheter balloon was used until the diameter of the septum was > 1 cm. Following the completion of endocardial ablation, electrodes and catheters were positioned around the rim of the fenestration in the upper, lower, anterior, and posterior regions through balloon atrial septostomy (Fig. 2) to verify that the fistula would not close spontaneously. Finally, electrodes, catheters, and balloons were removed.

#### Right Atrial Volume Overload

Sterile saline (0.9%) was administered to the pigs at a volume of 2000 mL daily for 1 month to induced the RA volume overload.

### Echocardiographic Protocol

The study used echocardiography to evaluate the hemodynamics of the right heart at baseline. A 2–4 MHz transducer was attached to a Vivid S6 ultrasound machine (GE Healthcare, USA). The American Society of Echocardiography standards were used to determine the severity of valvular insufficiency, with TR and mitral regurgitation (MR) graded as none or trace (0), mild (+ 1), moderate (+ 2), or severe (+ 3). Transthoracic echocardiography was used to monitor and navigate during surgery and to examine pericardial effusion postoperatively. The right and left cardiac parameters, including TR, tricuspid annulus diameter, tenting heigh, right atrium area (RAA), right ventricular inner diameter in systole (RVIDs), right ventricular inner diameter in diastole (RVIDd), left ventricular ejection fraction (LVEF), left atrial diameter (LAD), left ventricular end-diastolic dimension (LVDd), and mitral regurgitation (MR), were accessed by two-dimensional echocardiography with color flow Doppler at baseline and one-month post intervention.

### Autopsy

One month after treatment, the pigs were euthanized by administering a bolus of pentobarbitone at a dose of 150 mg/kg. The purpose of euthanasia was to enable the gross inspection of cardiac structures. The RA and RV were surgically exposed to thoroughly examine the tricuspid apparatus.

### Statistical Analyses

Continuous variables were presented as means ± standard deviations, whereas categorical data were expressed as percentages. The measured variables were compared between prior to initiation of intervention (Baseline) and one-month post intervention using paired Students’ two-tailed *t*-test for dependent observations with a *p*-value < 0.05 considered significant. Statistical analyses were conducted using Stata 15.1 software.

## Results

### Echocardiography

**Fig. 3:**
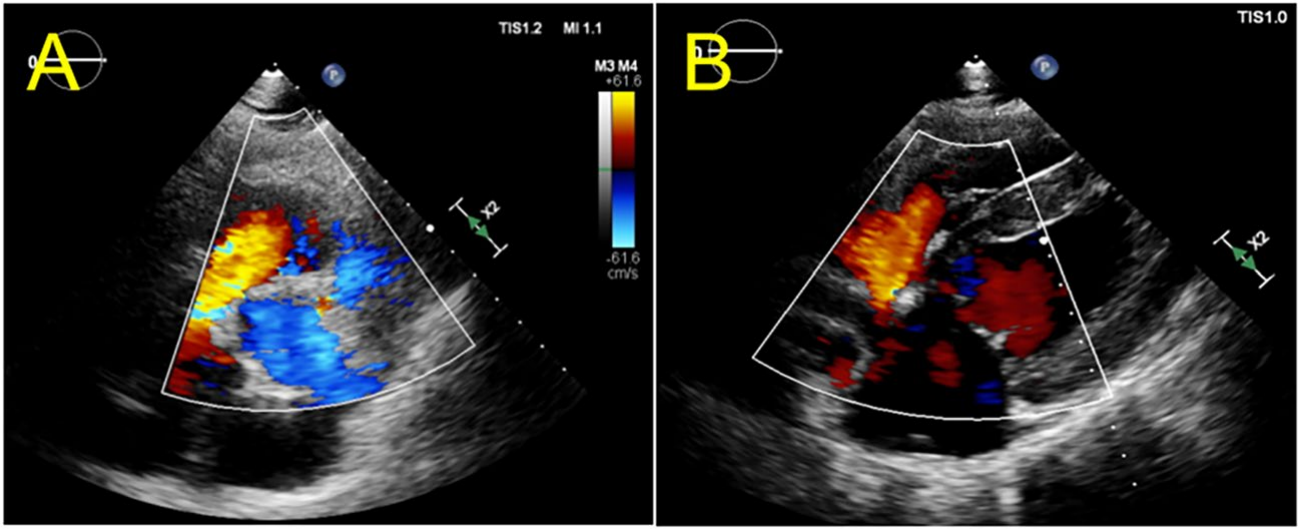
Color Doppler shows tricuspid regurgitation. **A, B** Tricuspid regurgitation at one-month post transcatheter procedures

Baseline echocardiographic examinations were performed on all swine subjects, revealing the normal anatomy of the RA and RV, with no indications of valve leaflet abnormalities, prolapse. The mean annular size measured 30.5 ± 4.2 mm, and only one subject has mild FTR during the initial observation period. Throughout the 1-month follow-up period, the swine subjects tolerated the development of TR well. The severity of FTR progressively increased during this period without leaflet rupture, as evidenced by echocardiographic examination (Fig. 3). Among the subjects, one (8.3%) exhibited severe FTR, eight (66.7%) exhibited moderate TR, and three (25%) exhibited mild FTR. The mean annular size in these subjects was significantly increased to 35.4 ± 4.0 (*p* = 0.008), with markedly increase of tenting height (6.7 ± 1.9 vs. 4.3 ± 1.8, *p* = 0.005). The enlargement of RA and RV was documented (RAA:152.5 ± 36.3 vs. 88.5 ± 17.6, *p* < 0.001; RVIDd: 32.5 ± 3.4 vs. 27.8 ± 2.3, *p* < 0.001; RVIDs: 25.6 ± 2.8 vs. 20.6 ± 2.1, *p* < 0.001). The increase in LAD, LVDd, and MR has no significant difference in one-month post operation comparing to those in baseline (Table 1).

**Table 1:** TTE characteristics in baseline and one-month post transcatheter procedures

Variable	Baseline line *N* = 12	1-month post transcatheter procedures *N* = 12	*p*
Mean TR grade 0 1 2 3	0.1 ± 0.1 11 (91.7%) 1 (8.3%) 0 0	1.8 ± 0.6 0 3 (25.0%) 8 (66.7%) 1 (8.3%)	< 0.001
Tenting heigh, mm	4.3 ± 1.8	6.7 ± 1.9	0.005
Tricuspid annulus diameter, mm	30.5 ± 4.2	35.4 ± 4.0	0.008
RAA, mm^2^	88.5 ± 17.6	152.5 ± 36.3	< 0.001
RVIDd, mm	27.8 ± 2.3	32.5 ± 3.4	< 0.001
RVIDs, mm	20.6 ± 2.1	25.6 ± 2.8	< 0.001
LVEF, %	69.3 ± 5.2	67.9 ± 4.9	0.50
LAD, mm	19.8 ± 4.8	20.4 ± 4.4	0.75
LVDd, mm	34.6 ± 3.2	35.2 ± 3.5	0.67
Mean MR grade 0 1 2 3	0.1 ± 0.1 11 (91.7%) 1 (8.3%) 0 0	0.2 ± 0.4 10 (83.3%) 2 (16.7%) 0 0	0.56

Data are presented as mean ± standard deviation or number (%) of patients

*TR* tricuspid regurgitation, *RAA* right atrium area, *RVIDs* right ventricular inner diameter in systole, *RVIDd* right ventricular inner diameter in diastole, *LVEF* left ventricular ejection fraction, *LAD* left atrial diameter, *LVDd* left ventricular end-diastolic dimension, *MR* mitral regurgitation

### TR Creation Procedure Details

**Fig. 4:**
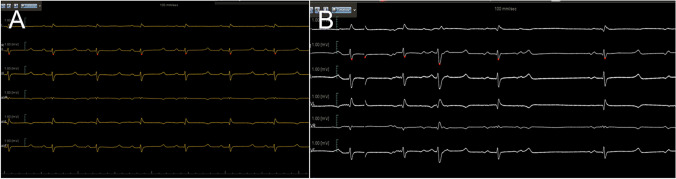
ECG monitoring system presents the rhythm. **A** Normal rhythm before ablation. **B** Premature atrial contraction post ablation

The mean duration of anesthesia in the swine subjects was recorded as 120 ± 10.7 min, while the operative time averaged 94.1 ± 9.4 min. Following this procedure, the ECG monitoring system detected an average of 510 ± 225 premature atrial contractions per hour (Fig. 4).

### Autopsy Findings

**Fig. 5:**
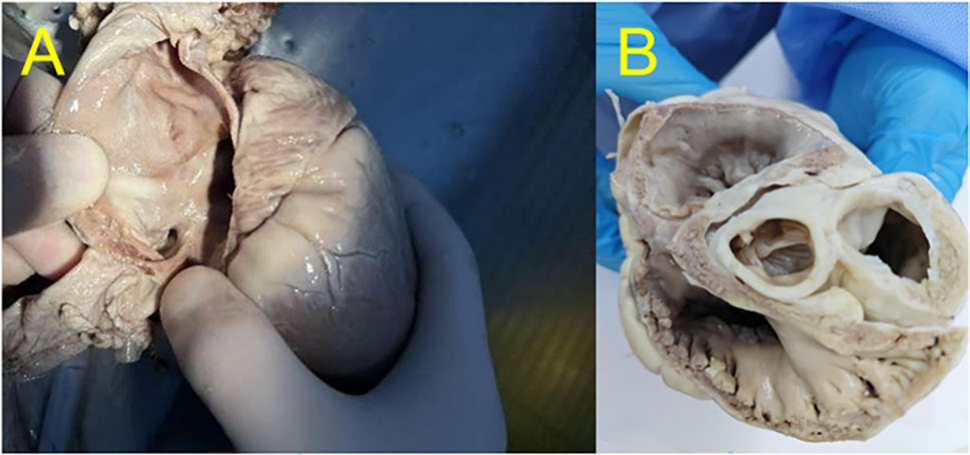
Autopsy shows the structure of right atrium. **A** The atrial septal defect.** B** The valve annulus

Upon gross examination of the enlarged heart, evidence of a left-to-right shunt in the atrial septum and tricuspid valve annulus enlargement was observed. However, no injury or damage was detected in the papillary muscles or anterior and posterior leaflets of the tricuspid valve. The mean atrial septal defect measured 1.5 ± 0.5 cm. Burn scars were observed in the endocardium of the RA and LA (Fig. 5).

## Discussion

In this study, we present a novel swine FTR model that uses a combination of radiofrequency ablation, atrial septostomy, and right atrial volume overload. Of the 12 pigs, one (8.3%) exhibited severe FTR, eight (66.7%) exhibited moderate TR, and three (25%) exhibited mild FTR after one month following the surgical procedure. Notably, the tricuspid annulus diameter, tenting height, RAA, and RV significantly increased compared with the preoperative measurements, highlighting the effectiveness and reproducibility of this innovative modeling approach.

Radiofrequency ablation was performed on the left and right endocardium to induce premature atrial beats in the swine subjects. During the study, all swine subjects experienced premature atrial contractions, with an average of 510 ± 225 occurrences per hour as monitored by an ECG. This can be attributed to scar formation in the atrium. A previous study reported that radiofrequency ablation creates deep and wide myocardial lesions when the electrode contacts the endocardium. This leads to contraction-band necrosis and subsequent edema, eventually becoming a scar [8]. The occurrence of heart rhythm disturbances is closely associated with the extent of myocardial injury; in this case, premature atrial contractions were triggered by endocardial ablation [9–11]. The persistence of premature atrial contractions subsequently results in hemodynamic disorders in both the left and right atria, leading to volume overload and the development of FTR.

A left-to-right shunt was created through transseptal puncture, radiofrequency ablation, and balloon dilation, allowing stable interatrial communication and blood flow from the LA to the RA. Radiofrequency ablation around the fenestration rim resulting from balloon atrial septostomy prevented spontaneous communication closure. Additionally, radiofrequency ablation of the fossa ovalis in our study reduced local elastic recoil. Using these methods, we established an artificial left-to-right shunt, which led to volume overload in the RA and subsequent enlargement of the RA, ultimately inducing TR. Patients with atrial septal defects are predisposed to FTR, and transcatheter atrial septal defect closure has been shown to significantly reduce FTR occurrence, which is consistent with the findings of previous studies [12–15]. Therefore, creating an artificial left-to-right shunt is a valuable approach to induce FTR in swine models [16]. Additionally, to the blood volume in the RA, sterile saline was administered to the swine subjects at a daily volume of 2000 mL over 1 month.

Using the composite measures described in this study, we successfully developed a valid and effective FTR model. Echocardiography performed one-month postoperatively confirmed the presence of TR in all swine subjects. Echocardiography and necropsy revealed a significantly larger tricuspid annulus, further supporting the establishment of a TR model. Importantly, our TR model was established through transcatheter intervention without mechanical disruption of the tricuspid valvular complex. Previous studies have employed transthoracic surgery or transcatheter intervention techniques that artificially damage the tricuspid valve complex, including the leaflets, papillary muscles, and tendon cords [17–19]. In contrast, our FTR model represents a more physiological approach, closely resembling the scenario observed for FTR. This distinction makes our model more realistic and clinically relevant for investigating TR in patients with FTR.

Previous studies have attempted to develop FTR models. Marcin et al. [6] used rapid ventricular pacing to induce FTR in ovine subjects. The procedure involved implanting a pacemaker with an epicardial left ventricular lead and placing sonomicrometry crystals on the right ventricle, along with telemetry pressure sensors on the left and right ventricles. The ovine subjects were paced at 220–240 beats/min until TR was observed. While this approach resulted in reliable and reproducible FTR models, it is important to note that the significant biventricular dysfunction and remodeling observed limits its applicability in reflecting the clinical condition of patients with end-stage heart failure who require mechanical support. In addition to in vivo models, researchers have developed in vitro FTR models. One such model is the swine heart bench model, which is considered to be a reliable system for simulating the pathophysiology of FTR [20]. In this method, a swine heart was mounted on a rigid support and immersed in a saline basin. A pump was used to convey saline from the basin to the right ventricle, thereby inducing FTR. This technique offers a simple and cost-effective approach for simulating FTR and can be used as a complementary approach for evaluating new technologies and therapies. Despite previous research, a viable in vivo model that accurately simulates atrial FTR in clinical settings remains to be established.

This study successfully established an atrial FTR model in swine subjects using a combination of catheter radiofrequency ablation, interatrial fistulation, and right atrial overload to induce right atrial dysfunction, which would cause right atrial myocardial fibrosis and cardiomyocyte hypertrophy, leading to RA enlargement [21]. Subsequently, an increase in the tricuspid annulus size and the development of TR were observed. Notably, many patients with FTR and atrial dysfunction also experienced atrial fibrillation and significant atrial enlargement [22, 23]. Therefore, our model successfully emulates the pathophysiology of FTR in this patient population, providing a reliable and effective platform for further research on simulating clinical FTR. By utilizing this model, we aimed to gain a deeper understanding of the underlying mechanisms involved in FTR and identify potential therapeutic targets for treating this condition.

This study has some limitations that must be acknowledged. First, the follow-up duration of the study may have been insufficient as it only spanned 1 month. More severe FTR would be observed with a longer follow-up duration. To address this potential limitation, we are conducting a study with a larger sample size and longer follow-up duration. Second, the development of FTR in our swine model required multiple treatments, including catheter radiofrequency ablation and interatrial fistulation. This complex approach requires a steep learning curve and high degree of expertise and experience on the part of operators. Finally, developing FTR models using these procedures incurs relatively high costs. This may limit the feasibility of the widespread use of our models; however, we believe that the benefits of developing accurate and reliable FTR models outweigh the cost limitations.

In conclusion, we successfully developed a novel swine FTR model using a combination of catheter radiofrequency ablation, interatrial fistulation, and right atrial volume overload. Our FTR model was demonstrated to be both effective and reliable, making it a valuable tool for studying the pathophysiology of FTR.

## Data Availability

The supporting data can be acquired via correspondence author.

## Abbreviations

TR: Tricuspid regurgitation
FTR: Functional tricuspid regurgitation
LVEF: Left ventricular ejection fraction
RA: Right atrium
RV: Right ventricle
RAA: Right atrium area
RVIDs: Right ventricular inner diameter in systole
RVIDd: Right ventricular inner diameter in diastole
LAD: Left atrial diameter
LVDd: Left ventricular end-diastolic dimension
MR: Mitral regurgitation
